# Prevalence and severity of anxiety, depression, and stress among optometry students in Nigeria: A cross-sectional study

**DOI:** 10.1371/journal.pone.0336195

**Published:** 2026-05-26

**Authors:** Michael Agyemang Kwarteng, Osamudiamen McHillary Ogiemudia, Bernadine N. Ekpenyong, Okechi U. Amaechi, Grace Ogbonna, Edith I. Daniel-Nwosu, Ngozika Esther Ezinne, Oforbuike Onyebuchi Ike, Kelechi C. Ogbuechi, Uchechukwu Levi Osuagwu

**Affiliations:** 1 Optometry Unit, Department of Clinical Surgical Sciences, Faculty of Medical Sciences, The University of the West Indies, St Augustine, Trinidad and Tobago; 2 Department of Environmental and Public Health Optometry, Faculty of Optometry, University of Benin, Benin City, Nigeria; 3 Department of Public Health, Faculty of Allied Medical Sciences, College of Medical Sciences, University of Calabar, Calabar, Cross River State, Nigeria; 4 Department of Optometry, Faculty of Health Sciences, Abia State University, Uturu, Abia State, Nigeria; 5 Department of Optometry, Faculty of Health Sciences, Mzuzu University, Mzuzu, Malawi; 6 Department of Optometry, Federal University of Technology Owerri, Owerri, Imo State, Nigeria; 7 Bathurst Rural Clinical School, School of Medicine, Western Sydney University, Campbelltown, Australia; 8 Department of Optometry, Bayero University Kano, Kano, Nigeria; 9 Department of Medicine, Dunedin School of Medicine, University of Otago, Dunedin, New Zealand; 10 Centre for Eyecare and Public Health Intervention Initiative (CEPHII), African Vision Research Institute, University of KwaZulu-Natal, Durban, South Africa; School of Nursing Sao Joao de Deus, Evora University, PORTUGAL

## Abstract

**Purpose:**

To assess the prevalence and severity of anxiety, depression, and stress among optometry students in Nigeria, and to identify demographic factors associated with mental distress.

**Methods:**

A cross-sectional, web-based survey was administered to optometry students from 10 Nigerian universities between 16 April and 18 November 2024, with an estimated sample size of 427. Mental health status was evaluated using the Depression, Anxiety, and Stress Scale-21 items (DASS-21). Collected data included age, gender, year of study, marital status, and DASS-21 scores. Descriptive statistics summarized demographic characteristics and the prevalence of symptoms. Binary logistic regression was used to identify associations between demographic variables and mental health outcomes, with significance set at p < 0.05.

**Results:**

A total of 474 students participated (mean age: 23 ± 3 years; 54.6% female; 94.1% unmarried). Overall, 51.1% experienced depressive symptoms, with 14.6% reporting severe or extremely severe symptoms. Severe anxiety was reported by 36.5%, and 25.1% experienced concurrent symptoms of anxiety, depression, and stress. Nearly half (49.2%) had at least two coexisting mental health conditions, indicating a significant emotional burden and potential impact on academic performance. Female students had significantly higher odds of experiencing depression (OR: 1.66; 95% CI:1.15–2.39) and overall mental distress (OR: 1.58; 95% CI: 1.03–2.43) compared to males.

**Conclusion:**

This study revealed a high prevalence of mental health conditions among Nigerian optometry students, with one in four experiencing a comorbid of all three conditions. Female students were more likely to report adverse mental health outcomes. These findings underscore the urgent need for structured mental health support and preventive interventions within optometry training programs in Nigeria.

## 1. Introduction

The global burden of mental health conditions, particularly depression, anxiety, and stress, continues to rise, significantly impacting quality of life and academic productivity [1]. Recent estimates indicate that one in eight individuals worldwide lives with a mental disorder, with anxiety and depression being the most prevalent [2]. While mental health among the general population has gained considerable attention, there is growing evidence of heightened vulnerability among university students, particularly those enrolled in high-demand health science programs [3,4].

Students in these disciplines face a unique set of stressors, including academic overload, clinical demands, examination pressure, and future career uncertainties [5–8]. These factors are known precipitating agents for psychological distress. However, within the health professional training landscape, optometry students have received relatively little research attention, especially in low- and middle-income countries like Nigeria, even though these professionals are a key segment within the health professional body [9].

Nigeria has seen a rapid expansion of its optometry program, growing from 3 to 14 tertiary institutions over the last four decades [10,11]. Currently, over 3,000 students are enrolled nationwide in a rigorous six-year Doctor of Optometry program that combines academic research with intensive clinical training [10]. Despite high enrolment, the program is often associated with significant failure and dropout rates, yet there remains a critical dearth of national data regarding the mental health outcomes of these students.

Preliminary evidence from related disciplines and single-centre studies in Nigeria suggests that health professional students face an elevated risk of burnout and psychological comorbidities [12,13]. Furthermore, recent cross-national research indicates that Nigerian university cohorts report among the highest levels of severe mental distress in Sub-Saharan Africa [14]. This highlights a systemic burden within Nigerian higher education that requires targeted, discipline-specific investigation.

Beyond academic stressors, Nigerian students navigate unique challenges arising from socio-political instability and financial hardship. Frequent industrial actions, most notably by the Academic Staff Union of Universities (ASUU), lead to prolonged university closures and disrupted academic calendars [15,16]. These disruptions, combined with deteriorating infrastructure and campus insecurity, contribute significantly to student anxiety and academic burnout.

Additionally, the COVID-19 pandemic intensified existing challenges by introducing online learning fatigue and interrupting essential clinical training [17,18]. In the Nigerian context, these stressors are often compounded by the stigma surrounding mental health, which frequently acts as a barrier to help-seeking behaviour and social support [19].

The present study addresses a critical gap in the literature by providing evidence-based insights into the prevalence and severity of mental health conditions among Nigerian optometry students. Utilising the standardised Depression, Anxiety, and Stress Scale-21 items (DASS-21), this research seeks to identify socio-demographic determinants of distress. The findings aim to inform the development of targeted mental health policies and wellness strategies, aligning with the WHO’s Comprehensive Mental Health Action Plan to expand services in youth mental health [20].

## 2. Subjects and methods

### 2.1. Participants

This study focused exclusively on optometry students enrolled in optometry schools in Nigeria. Participants were recruited from institutions offering optometry programs, and the survey targeted individuals currently pursuing undergraduate optometry degrees in Nigeria.

### 2.2. Setting

Nigeria, the most populous country in Africa, had a gross domestic product (GDP) of $252.7 billion in 2022 and a per capita GDP of $1,100 [21]. As a key Sub-Saharan African (SSA) country with a growing higher education sector, Nigeria provides a relevant setting for exploring the mental health status of optometry students. There are 14 optometry schools in Nigeria [10].

### 2.3. Study design and procedure

A web-based, cross-sectional survey was conducted using a convenience sampling approach from Tuesday, April 16th to Monday, November 18th, 2024. A validated, self-administered questionnaire was distributed in English via Google Forms. The survey link was shared through social media platforms (e.g., WhatsApp, Facebook) and institutional email networks, using a snowballing strategy to enhance reach.

All prospective participants received a brief online description of the study’s purpose and procedures before proceeding. The survey strictly followed the STROBE guidelines for cross-sectional studies [22].

### 2.4. Questionnaire

A validated, self-administered survey [23] was adapted to suit the study’s objectives. The first section collected sociodemographic data, including age, gender, university, year of study, and marital status.

#### 2.4.1. DASS-21 survey scale.

The Depression, Anxiety, and Stress Scale-21 (DASS-21) is a well-established, psychometrically robust instrument used globally to measure the severity of these three negative emotional states [23]. It enables researchers to examine mental health status with internal validity and cross-cultural adaptability [24]. The DASS-21 scale has been validated in several developing country contexts and is appropriate for assessing student mental health in Nigeria [25]. This instrument contains 21 items, divided equally across three subscales (depression, anxiety, stress), with responses on a 4-point Likert scale (0 = did not apply at all; 3 = applied very much). The classification thresholds were:

- Depression: 0–9 (normal), 10–13 (mild), 14–20 (moderate), 21–27 (severe), 28+ (extremely severe)
- Anxiety: 0–7 (normal), 8–9 (mild), 10–14 (moderate), 15–19 (severe), 20+ (extremely severe)
- Stress: 0–14 (normal), 15–18 (mild), 19–25 (moderate), 26–33 (severe), 34+ (extremely severe)

The questionnaire was pre-tested with 10 optometry students from a Nigerian university one week before the main study. Feedback was used to refine the tool. The Cronbach’s alpha coefficient for the DASS-21 in this cohort was 0.82, indicating good internal consistency.

### 2.5. Inclusion and exclusion criteria

Only responses from undergraduate optometry students currently enrolled in Nigerian universities were included. Participants had to provide informed consent to participate. Duplicate entries, identified through identical IP addresses and matching demographics, were filtered, and only the most complete entry was retained.

### 2.6. Sample size determination

The required sample size was calculated using the single-proportion formula for cross-sectional studies:

$n=z^{\text{2}}P(\text{1}-P)/d^{\text{2}}$ [27]

where *n* is the minimum sample size, *Z* is the standard normal deviate corresponding to the desired confidence level, *P* is the estimated prevalence, and *d* is the margin of error. A 95% confidence level (*Z* = 1.96), 5% precision (*d* = 0.05), and an estimated prevalence of 50% were used. The prevalence was set at 50% due to the absence of prior studies assessing mental health symptoms in this population, which provides the maximum sample size [26]. This yielded a minimum required sample size of 384 participants. After adjusting for a 10% non-response rate, the final calculated sample size was 427 participants. A total of 474 valid responses were obtained and included in the final analysis.

#### 2.6.1. Reliability test.

The DASS-21 demonstrated strong reliability in this population. The overall Cronbach’s alpha was 0.942. The subscales for: Depression: α = 0.806, Anxiety: α = 0.873 and Stress: α = 0.871. Each subscale included 7 items.

### 2.7. Data analysis

The DASS-21 scores were analysed as categorical variables. Mental health symptoms were dichotomised for analysis:

- Depression: 0–9 = No depression; 10+ = depression
- Anxiety: 0–7 = No anxiety; 8+ = Anxiety
- Stress: 0–14 = No stress; 15+ = Stress

Sociodemographic factors included age, gender, marital status, level of study, and school affiliation. Frequencies and percentages were used to summarise sample characteristics. Prevalence rates were estimated using binomial distributions with Clopper-Pearson confidence intervals (CI). Bivariate analyses were used to examine associations between mental health outcomes and sociodemographic characteristics. Subsequently, multivariable logistic regression was conducted, adjusting for age, gender, marital status, level of study, and university. Results of the associations were reported using unadjusted odds ratios (OR) and adjusted odds ratios (AOR) with their CIs. Only variables that reached a significance level of *P* < 0.20 in the bivariate analysis were included in the multivariable logistic regression model to maintain model parsimony. A p-value < 0.05 was considered statistically significant. Analyses were conducted using IBM SPSS v21.0 and R v4.0.3.

### 2.8. Ethical considerations

Ethical approval was obtained from Nigerian institutional ethics boards, including Federal University of Technology Owerri, Nigeria (FUT/SOHT/REC/vol. 4/2), Abia State University, Uturu, Nigeria (ABSU/REC/OPT/002/2024), and Bayero University Kano, Nigeria (NHREC/BUK-HREC/476/10/2311). Consent was obtained through a mandatory electronic checkbox, which constitutes electronic written consent. For this, the participants were required to select “Yes” to the question: *“Do you consent to voluntarily participate in this survey?”* Participants could not proceed without consenting.

## 3. Results

**Fig 1:**
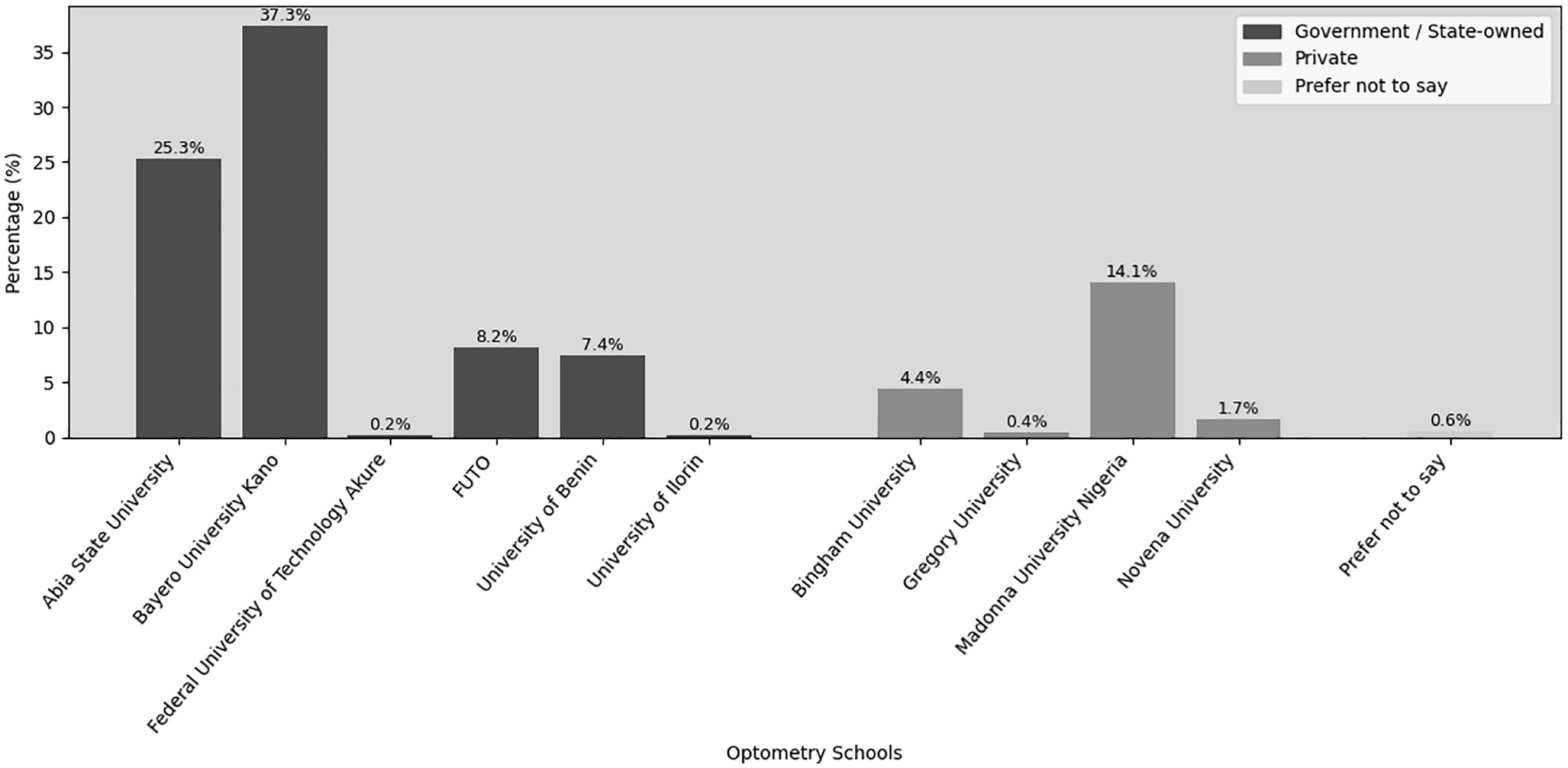
Distribution of participants by their institutions of learning. FUTO = Federal University of Technology.

A total of 474 participants from 10 universities were involved in this study (see Fig 1). Government-owned institutions accounted for the majority of respondents (78.6%), with Bayero University (37.3%) and Abia State University (25.3%) contributing the largest proportions. Private institutions represented 20.6% of respondents, primarily from Madonna University (14.1%). A small proportion of respondents (0.6%) preferred not to disclose their institution.

The age of the participants ranged from 17 to 42 years (mean: 23 ± 3 years), mostly female (54.6%), not married (94.1%), and Christianity was the predominant religion (62.7%). In terms of year of study, 70.1% were in the clinical year, while 29.9% were in the preclinical year (years 1–4). Most respondents (95.1%) reported not having children, with only 4.9% indicating that they did, Table 1.

**Table 1:** Distribution of demographic characteristics.

Variable	Category	Frequency (n)	Percentage (%)
Age group (years)	Younger age (<18yrs)	5	1.1
	Youth (18–35yrs)	467	98.5
	Adult (≥36yrs)	2	0.4
Gender	Female	259	54.6
	Male	215	45.4
Ethnicity	African descent	467	98.5
	Others	7	1.5
Marital status	Married/relationship	28	5.9
	Not married	446	94.1
Religion	Christianity	297	62.7
	Islamic	170	35.9
	Others	7	1.5
Year of study (n = 465)^a^	Preclinical (Year 1–4)	139	29.9
	Clinical (Year 5–6)	326	70.1
Have Children	Yes	23	4.9
	No	451	95.1

a n=465 are those that responded to the “Year of study”

### 3.1. Distribution of the severity of mental health conditions

**Fig 2:**
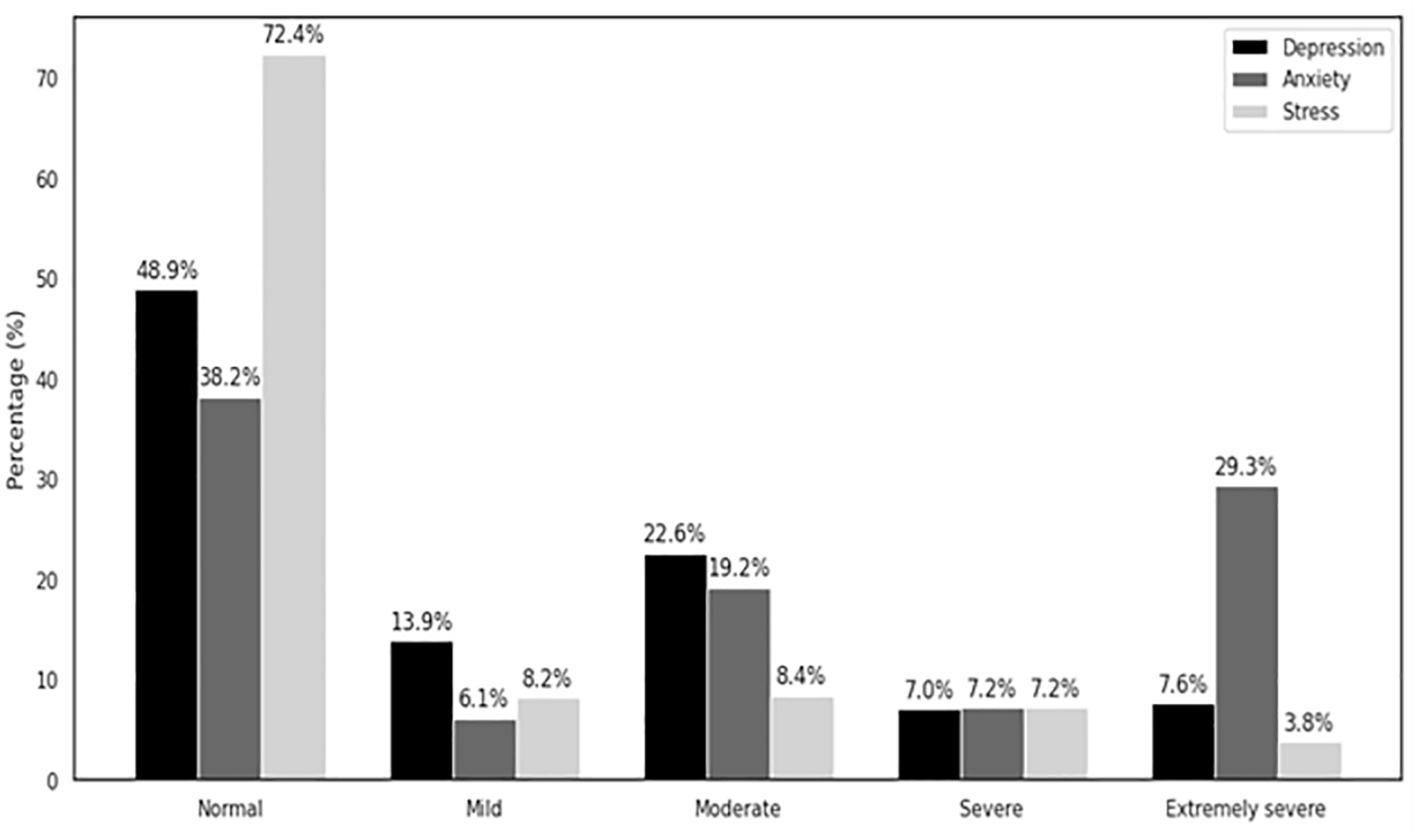
Distribution of the severity of mental health conditions.

Figure 2 illustrates the severity distribution of anxiety, depression, and stress. Anxiety showed a notably high proportion of participants in the severe and extremely severe categories combined, totalling 36.5% (7.2% severe and 29.3% extremely severe), which is substantially higher compared to depression (14.6%) and stress (11.0%) in the same combined categories.

### 3.2. Distribution of the prevalence of mental health conditions

**Fig 3:**
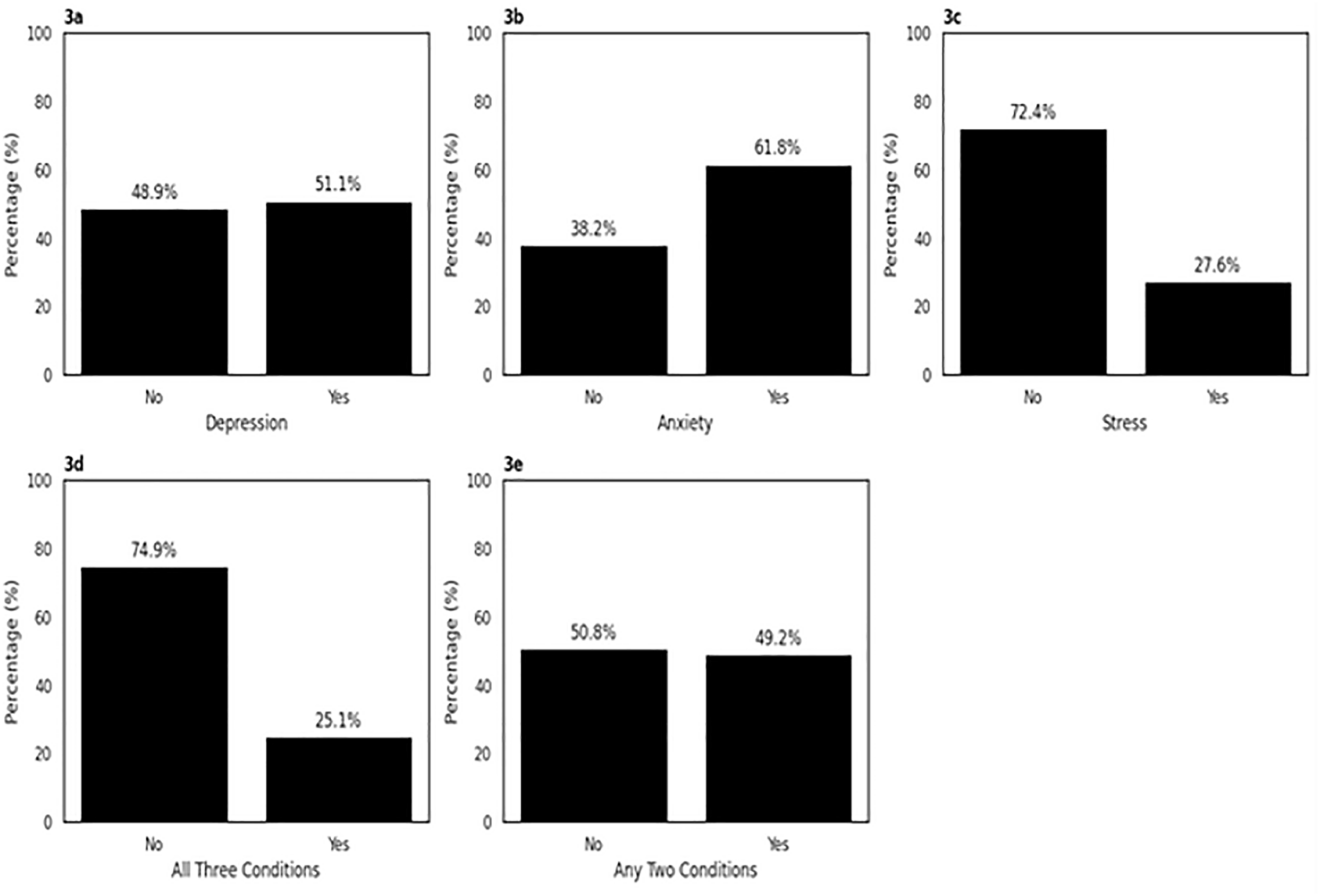
Distribution of the prevalence of mental health conditions.

Over half of the participants reported experiencing a form of depression (n = 242, 51.1%) and anxiety (n = 293, 61.8%), while a smaller proportion reported stress (n = 131, 27.6%), Fig 3. Additionally, 25.1% of participants experienced all three conditions concurrently, and 49.2% experienced any two of these mental health issues. The prevalence of anxiety was notably higher compared to depression and stress, indicating a significant mental health burden.

### 3.3. Binary logistic regression analysis of mental health conditions and demographics

A binary logistic regression was conducted to assess whether demographics predict mental health status. With each additional year of age, the odds of reporting symptoms of depression, anxiety, stress, or their co-occurrence decreased significantly, with odds ratios ranging from 0.91 to 0.93, Table 2. Female students had higher odds of depression (OR=1.66, 95% CI: 1.15–2.39) and experiencing all three mental health issues (OR=1.58, 95% CI: 1.03–2.43) compared to males. Being married was linked to lower odds of anxiety (OR=0.44, 95% CI: 0.20–0.95). Preclinical students, compared to clinical students, had higher odds of experiencing stress (OR = 1.59, 95% CI: 1.04–2.45), any two mental health issues (OR = 1.55, 95% CI: 1.04–2.31), and all three issues combined (OR = 1.61, 95% CI: 1.03–2.50), Table 2.

**Table 2:** Binary logistic regression analysis of mental health conditions and demographics.

Variable	Reference category	Dependent variable	Odds ratio (95% CI)	Adjusted ORs
Age	Continuous variable	Depression	**0.92 (0.87-0.98)**	0.94 (0.88-1.01)
		Anxiety	**0.93 (0.88-0.98)**	**0.93 (0.87-0.99)**
		Stress	**0.91 (0.85-0.97)**	**0.92 (0.86-0.99)**
		Any two	**0.92 (0.87-0.98)**	**0.93 (0.87-0.99)**
		All three	**0.91 (0.85-0.98)**	0.94 (0.88-1.00)
Gender (female)	Male	Depression	**1.66 (1.15-2.39)**	1.45 (0.99-2.14)
		Anxiety	1.15 (0.79-1.67)	–
		Stress	1.44 (0.85-2.17)	–
		Any two	1.35 (0.94-1.93)	–
		All three	**1.58 (1.03-2.43)**	1.34 (0.85-2.11)
Marital status (married/relationship)	Not married	Depression	0.51 (0.23-1.14)	–
		Anxiety	**0.44 (0.20-0.95)**	0.61 (0.26-1.41)
		Stress	0.55 (0.21-1.48)	–
		Any two	0.47 (0.21-1.06)	–
		All three	0.63 (0.24-1.70)	–
Having Children (No)	Yes	Depression	2.02 (0.84-4.86)	–
		Anxiety	1.52 (0.65-3.51)	–
		Stress	1.86 (0.62-5.58)	–
		Any two	2.29 (0.93-5.69)	–
		All three	1.63 (0.54-4.88)	–
Year of Study (Preclinical students)	Clinical students	Depression	1.44 (0.97-2.15)	–
		Anxiety	1.39 (0.92-2.11)	–
		Stress	**1.59 (1.04-2.45)**	1.25 (0.77-2.05)
		Any two	**1.55 (1.04-2.31)**	1.24 (0.79-1.94)
		All three	**1.61 (1.03-2.50)**	1.26 (0.76-2.09)

Odd ratios (OR) with Confidence intervals (CI) that exclude 1.00 are statistically significant, and bolded show statistically significant. Only variables that reached a significance level of *P < 0.05* in the bivariate analysis were included in the multivariable logistic regression model to maintain model parsimony.

After adjusting for significant variables such as age, year of study and marital status, the association between gender and depression attenuated, with the odds of depression among female students decreasing from an unadjusted OR of 1.66 (95% CI: 1.15–2.39) to an adjusted OR of 1.45 (95% CI: 0.99–2.14), rendering the association statistically non-significant. Similarly, the odds of experiencing all three mental health conditions among females declined from 1.58 (95% CI: 1.03–2.43) to AOR of 1.34 (95% CI: 0.85–2.11). Marital status was no longer a significant predictor of anxiety, with an adjusted OR of 0.61 (95% CI: 0.26–1.41). For year of study, clinical students initially showed higher odds of stress (OR = 1.59), any two conditions (OR = 1.55), and all three conditions (OR = 1.61); however, after adjusting for age, these associations weakened and were no longer significant (AORs = 1.25, 1.24, and 1.26, respectively).

## 4. Discussion

A significant mental health burden among this population was highlighted by examining the prevalence of anxiety, depression, and stress among university students in this study. The findings indicate a high prevalence of mental health challenges, especially anxiety, and emphasize key demographic factors influencing mental health outcomes. Anxiety emerged as the most common and severe mental health issue among participants, with a notable portion (61.8%, n = 293) reporting symptoms and over one-third (36.5%, n = 173) experiencing them at severe to extremely severe levels. This high prevalence highlights anxiety as a major mental health concern within the study population. These results align with previous studies [27–30], which also noted considerable variation in the severity of mental health symptoms, with anxiety often reported as the most prominent. Furthermore, to illustrate the scope of the problem, it has been reported that higher levels of depression, anxiety, and stress are closely linked to both suicidal ideation and academic underperformance in university students [31,32].

The study also showed the co-occurrence of mental health conditions in the population. A quarter of participants (25.1%) experienced anxiety, depression, and stress concurrently, and nearly half (49.2%) experienced any two conditions. This clustering of mental health conditions is consistent with the finding [33], which indicates a strong positive correlation among the three conditions, where an increase in one symptom is closely associated with significant increases in the others.

The significant demographic predictors of mental health outcomes in the current study were age, gender, and marital status. This study identified age as a significant protective factor against mental health challenges, with older students consistently showing lower odds of experiencing depression, anxiety, stress, or their co-occurrence (Table 2). Regarding gender, female students had significantly higher odds of experiencing depression and all three conditions concurrently. This finding aligns with previous research works [34] but differs from other studies that reported higher levels of mental health conditions in male students [35,36] while Othman et al. [37] found no gender based differences. The study also highlighted the influence of marital status on mental health outcomes among university students. Consistent with previous research, results from the current study showed that married students or those with close relations are less likely to experience anxiety compared with those without close relations. This may reflect marital closeness or close relation, which provides some emotional and psychological stability, safety, and protection. Also, it aligns with a report of a study [38] that showed lower levels of psychosocial distress among students with close relation due to role of social support in the relationship mitigating the psychosocial distress.

The potential influence of sociocultural factors and gender-related expectations within the Nigerian context warrants consideration. It is plausible that unmarried students, particularly females, may encounter societal pressures regarding marital timing or domestic caregiving responsibilities. Such factors could contribute to heightened levels of anxiety or academic stress; however, as these variables were not explicitly measured in the current study, these interpretations remain speculative. Further qualitative research is required to substantiate the impact of these specific sociocultural determinants on the mental well-being of optometry students.

Regarding the year of study, findings revealed that students in their preclinical years were 1.6 times more likely to experience stress, comorbidity of any two conditions, and the presence of all three mental health conditions increased significantly than those in the clinical years. This may be due to preclinical students facing greater adjustment challenges, heavier theoretical coursework, and unfamiliar academic demands during the early years of training, which can increase stress levels and the risk of multiple mental health difficulties compared to students who have progressed to clinical years and may have developed better coping strategies and support networks. The findings of this study is consistent with other studies in which students in pre-clinical years had higher depression, anxiety, and stress scores than those in clinical years, but this differs from that observed in another research [39] that reported contrasting findings. Also, another study found that the year of study had no significant effect on outcome measures in mental health [40].

### 4.1. Strengths and limitations

The study focused on the levels of mental health conditions among optometry students in Nigeria. The homogeneity within the sample supports more controlled analyses of variable factors such as marital status and year of study. The validated DASS-21 scale ensured that the data were reliable, and the web-based survey enabled widespread reach. The use of convenience and snowball sampling via social media, may lead to substantial selection bias. While these methods were utilised to maximise reach across 10 universities during periods of potential academic disruption, we recognise they may introduce selection bias. Students with a greater interest in mental health or those experiencing higher levels of distress might have been more likely to participate, potentially inflating prevalence estimates. This may limit the generalizability of the study findings to the broader population of Nigerian students. Optometry students in Nigeria largely belong to the relatively younger population, and this may affect how they experience and report mental health issues. The cross-sectional design limits the establishment of the cause-and-effect relationship of factors. Furthermore, the use of self-reported data introduces potential bias, especially given the significant stigma surrounding mental health in Nigerian society. Future research could benefit from incorporating qualitative data, such as responses to open-ended survey questions, to gain richer insights into respondents’ mental health-related beliefs and real-life experiences.

### 4.2. Conclusions

This study revealed a substantial burden of mental health conditions among Nigerian optometry students, with anxiety being the most prevalent. A significant proportion of students experience multiple co-occurring conditions, with one in four reporting concurrent anxiety, depression, and stress. Female students and those in preclinical years were identified as high-risk groups, while being older or married appeared to be protective. The findings underscore the need for integrated institutional responses, including the incorporation of mental health education into optometry curricula, the provision of accessible and confidential counselling services, and the development of targeted support strategies for high-risk groups. National regulatory bodies, such as the Optometrists and Dispensing Opticians Registration Board of Nigeria (ODORBN), should consider developing standardized mental health guidelines for training institutions. Additionally, inter-professional collaboration, improved infrastructure, and policy interventions addressing systemic contributors to student distress, such as academic instability and resource limitations, are essential. Public health authorities should prioritize student mental health in national strategies and implement evidence-based awareness and stigma-reduction campaigns. Finally, sustainable funding and support for longitudinal research are necessary to monitor trends, evaluate interventions, and inform policy and practice across tertiary institutions.

## Supporting information

Supporting information file Data for Optometry students’ mental health in Nigeria. (XLSX)
